# Do the pollution related to high-traffic roads in urbanised areas pose a significant threat to the local population?

**DOI:** 10.1007/s10661-016-5697-1

**Published:** 2016-12-24

**Authors:** Joanna Kobza, Mariusz Geremek

**Affiliations:** Public Health Department, School of Public Health, Medical University of Silesia in Katowice, Piekarska 18, 41-902 Bytom, Poland

**Keywords:** Traffic-related air pollution, Population health, Nitrogen dioxide, Carbon monoxide, Environmental policy

## Abstract

Many large neighbourhoods are located near heavy-traffic roads; therefore, it is necessary to control the levels of air pollution near road exposure. The primary air pollutants emitted by motor vehicles are CO, NO_2_ and PM. Various investigations identify key health outcomes to be consistently associated with NO_2_ and CO. The objective of this study was the measurement-based assessment for determining whether by high-traffic roads, such as motorways and express ways, and the concentrations of CO and NO_2_ are within normal limits and do not pose threat to the local population. Average daily values (arithmetic values calculated for 1-h values within 24 h or less, depending on result availability) were measured for concentrations of NO_2_ and CO by automatic stations belonging to the Voivodship Environmental Protection Inspectorate in Katowice, in areas with similar dominant source of pollutant emission. The measurements were made in three sites: near the motorway and expressway, where the average daily traffic intensity is 100983 and 35414 of vehicles relatively. No evidence was found of exceeding average daily values equal to the maximum allowable NO_2_ concentration due to the protection of human health in the measurement area of the stations. No daily average values exceeding the admissible CO concentration (8-h moving average) were noted in the examined period. The results clearly show lack of hazards for general population health in terms of increased concentrations of CO and NO_2_ compounds that are closely related to high intensity car traffic found on selected motorways and speedways located near the city centres.

## Introduction

Air quality and its impact on health are major environmental health issues (WHO 2012). Vehicular exhaust has become a main source of air pollution in urbanised areas. The primary air pollutants emitted by motor vehicles are carbon monoxide (CO), nitrogen oxides (NO_x_ including NO and NO_2_) and particulate matter (PM). Numerous epidemiological studies supported by some toxicological investigations demonstrate a positive association between NO_2_ and CO concentration and increased adverse respiratory and cardiovascular events, including morbidity and mortality (Anderson, 2009; Samoli et al., 2005; Ostro et al., 2009; Fenger, 2009; Bérard et al., 2015). Even short exposure to diesel exhaust fumes found in the air in concentrations that are typical of areas close to high-traffic roads, in urbanised areas, has an effect on vasoconstriction (narrowing of the lumen of vessels resulting from contraction of smooth muscular coat), thus leading to increased arterial blood pressure or to decreased local blood flow; what is also observed is increased rigidity of blood vessels as a result of concomitant atherosclerosis, increased blood clotting capability (through inhibition of fibrinolytic factor secretion) and myocardial ischaemia (Lucking et al., 2008, Lundbäck et al., 2009; Mills et al., 2007; Mills, 2005; Peretz et al., 2008). Increased NO_2_ level and presence of PM_2.5_ particles in the atmosphere may also be related to the risk of increased cholesterol level (Sørensen et al., 2015). Many hours of staying in places of residence located near high-traffic roads is connected with increased level of the AC133+ stem cells responsible for processes of blood vessel reparation, the presence of which in peripheral blood may be an early indicator of damage to the cardiovascular system (caused to a high extent by particles with PM_2.5_ diameter) (De Jarnett et al., 2015). Exposure to noise and air pollution may also lead to elevated peripheral blood pressure and increase the risk of cardiovascular diseases (Chang et al., 2015; Miller et al., 2015; Fecht et al., 2016). Constant remaining in locations situated near high-traffic roads is also connected with the risk of increased CRP (C-reactive protein) concentration in peripheral blood, which is an important risk factor of CVD (cardiovascular diseases) (Lanki et al., 2015). Part of studies showed a relationship between a long-lasting exposure of residents to increased NO_2_ level and death rate due to coronary artery disease (Gan et al., 2011). A correlation was also demonstrated between elevated CO concentration and increased number of admissions of adults due to CVD and of children due to respiratory diseases (Samoli et al., 2016). Based on the recent studies, elevated CO and NO_2_ concentrations have a considerable effect on the number of patients reporting to Emergency Departments in hospitals due to exacerbation of asthma (Zheng et al., 2015; Khamutian et al., 2015). Significantly increased levels of NO_2_, CO and PM_2.5_ particles in the atmosphere also lead to increased risk of premature births, possibility of hypertension in pregnant women and risk of preeclampsia (Pedersen et al., 2014; Männistö et al., 2015; Li et al., 2016; Qian et al., 2016).

Nitrogen oxides rank among the most dangerous compounds. They get into the atmosphere through human activity. They play a significant role in the formation of such adverse events as acid rains, winter smog and photochemical smog and indirectly—they act as a tropospheric ozone precursor. NO_2_ is a brutal gas with sharp odour. It is produced, for instance, as a result of nitrogen oxide (NO) oxidation. It is strongly toxic for the respiratory and immune systems in the human body. As a result of the oxidation of nitrogen found in the air and high temperatures occurring in the processes of combustion, nitrogen oxide is produced: N_2_ + O_2_ = 2 NO. As the temperature drops, nitrogen oxide becomes nitrogen dioxide along the following formula: 2 NO + O_2_ = 2NO_2_, and is usually emitted into the atmosphere in this form. In the polluted atmosphere and under the influence of ozone or hydroxyl radical, NO_2_ oxidises to nitrogen acid (HNO_3_). Ions produced as a result of nitrogen acid dissociation are the major acidifying compounds. Moreover, nitrogen ion NO_3_- has a considerable effect on eutrophication of terrestrial and aqueous ecosystems. Nitrates produced upon binding with other compounds found in the atmosphere, e.g. ammonia, become an important factor facilitating the formation of the PM_2.5_ dust particles (Ekoportal, 2016; Government of Canada, 2010).

Carbon oxide is an inflammable gas, where carbon occurs in the 2nd oxidation degree. In the atmosphere, it oxidises to CO_2_, which results in the production of ozone. CO is a colourless and odourless gas strongly toxic for the human body, which—due to low specific gravity—spreads in the atmospheric air quickly. The main source of CO emission is road transport, petrol engine-driven vehicles in particular. Anthropogenic emission is first of all caused by fuel combustion processes—CO is produced upon incomplete combustion of carbon. The volume of emission depends on the type of vehicle, its speed and operating principle (Ekoportal, 2016). In concentration higher than 5000 ppm, its adverse effect on the human body is evident.

### Aim

The objective of this study was the measurement-based assessment for determining whether by high-traffic roads, such as motorways and express ways, the concentrations of CO and NO_2_ are within normal limits and do not pose threat to the local population.

### Methodology

The concentrations of CO and NO_2_ in air were measured. The data used in this study were obtained from three automatic stations of the State Environmental Monitoring in the Silesian Voivodship (Upper Silesian urban area), with dominant source of pollutant emission with car communication background. The measurements were made in years 2007–2014 in three sites: Chorzów Batory, near the A4 motorway (longitude 18°56′15″ E, latitude 50°15′15″ N); Katowice at Górnośląska Avenue (longitude 19°1′10.20″ E, latitude 50°14′48.50″ N) and Częstochowa at Armii Krajowej Street (longitude 19°7′2.70″ E, latitude 50°49′3.65″ N). All measurement stations belong to the Voivodship Environmental Protection Inspectorate (VEPI) in Katowice. Average daily values (arithmetic values calculated for 1-h values within 24 h or less) were measured for concentrations of NO_2_ and CO. Although analysed NO_2_ and CO concentrations within 24 h show asymmetry, the studies employed the arithmetic average as this is the only average measure (for CO, arithmetic moving average for the interval of 8 h) calculated in the analysis of air pollutant concentrations. Additionally, an analysis was conducted of 1-h values of NO_2_ and maximal 8-h averages—from among moving averages—for CO in the relevant periods of time so as to assess the statistical probability of the studied air pollutant concentrations being higher than maximum allowable, due to protection of human health. The statistical analyses were performed by means of Stat Soft, Inc. (2011) STATISTICA version 10, whereas other calculations were carried out using the Microsoft Excel 2010 spreadsheet. The dependence was studied with correlation coefficient γ in ordinal scale and with Kendall rank correlation coefficient τ(τK) in ratio scale. A correlation between classes of air index and years for selected measurement stations was analysed with Cramer’s V. Empirical distribution of NO_2_ and CO concentrations in the studied periods of time were surveyed with association (contingency) tables or with use of graphic form, i.e. categorised histograms. Changes in the largest values of arithmetic average of NO_2_ and CO concentrations in the studied periods of time, in particular months, were shown with charts with polar coordinates (radar charts), where months in consecutive studied years were determined with a triangle while values of air pollutant concentrations were reflected by the distance from the centre (beginning) of the coordinate system.

## Results

### Common air quality index

The analysis of air quality in the world uses many indicators. One of those is CAQI (Common Air Quality Index), which is used for classification and comparison of air quality in European Union states. It is also employed by the EEA (European Environmental Agency) to provide information on the environment. The comparison of air quality in selected cities is based on two categories of measurement stations: communication stations and urban background stations. CAQI is calculated for three basic pollutants: PM_10_, NO_2_, O_3_ and, additionally, for PM_2,5_, CO and SO_2_. The above air quality index has got five categories (classes, levels). Classes 1 to 3 reflect satisfactory air quality, i.e. the air pollution level that does not pose threat to human health, while Classes 4 and 5 reflect bad air quality, i.e. the air pollution level that does pose threat to human health, particularly in the so-called high-risk groups (Class 4) or the general population (Class 5) (Table 1).

**Table 1:** Recommended actions in case of occurrence of the given air quality index class, based on the U.S. Environmental Protection Agency (EPA) recommendations

Air quality index/classes	Air pollution level	General population	High-risk population
Class 1 Class 2	Very low Low	Satisfactory air quality, no or low health risk due to air pollution. It is possible to stay and perform any activities outdoors	Satisfactory air quality, no or low health risk due to air pollution. It is possible to stay and perform any activities outdoors
Class 3	Average	The regular tasks outdoors do not have to be changed	It is necessary to consider decrease or distribution of outdoor activity over time if it requires long-term or intensified effort, especially in case of any aggravated health condition symptoms
Class 4	High	It is necessary to consider decrease or distribution of outdoor activity over time if it requires long-term or intensified effort	Outdoor activity which requires long-term or intensified effort must be limited and it is necessary to consider leaving children, elderly persons, pregnant women and persons with diagnosed respiratory or circulatory system diseases at home
Class 5	Very high	It is recommended to minimise any activities outdoor, especially if they require long-term or intensified effort	Children, elderly persons, pregnant women and persons with diagnosed respiratory or circulatory system diseases should stay at home

### National and European policy

ln Poland, the maximum allowable level of average annual concentration of NO_2_ is 40 μg/m^3^ while the maximum allowable level of annual 1-h concentration is 200 μg/m^3^. This level can be exceeded up to 18 times per year. The alarm level of average 1-h concentration is 200 μg/m^3^ (Journal of Laws 2002a, No. 87, item 796). The maximum allowable concentration of CO in the air is 10,000 μg/m^3^ (J. Laws 2012, No.217, item 1031). In the outdoor air, CO concentration is ca. 300–350 ppm in cities and 400 ppm in industrial areas. Pursuant to the Regulation (Journal of Laws (2002b), no. 217, item 1833, threshold limit value (TLV) of carbon oxide in the working environment is 23 μg/m^3^ and short-term TLV is 117 μg/m^3^. Furthermore, in Poland, the maximum allowable carbon oxide level in the outdoor air is 10,000 μg/m^3^ (Journal of Laws, No. 87, item 796) (measurement result averaging time—8 h) and in the indoor air of health service, its concentration should not exceed 30 μg/m^3^ within 24 h and 100 μg/m^3^ within 30 min (J. Laws 2008, No.47, item 281) (Table 2).

**Table 2:** The methods of indexing, averaging times and the intervals of air pollutant concentrations of air quality index for Silesian Voivodship. The maximum allowable and alarm concentrations for NO_2_ and CO in air

Air quality index	The concentration of NO_2_ (μg/m^3^)	The concentration of CO (μg/m^3^)
	Averaging times	Averaging times
	1 h	8-h moving average
Class 1 Very low	0–50	0–5000
Class 2 Low	51–100	5000–7500
Class 3 Average	101–200	7500–10,000
Class 4 High	200–400	10,000–20,000
Class 5 Very high	>400	>20,000
Maximum allowable level	1 h—200 1 year—40	8 h—10,000
Alarm level	1 h—400	–

The EC Directive 2008/50/EC on ambient air quality (EC, 2008) limits air pollution to levels that minimise its adverse effects on population health and environment. It determines the quality standards of ambient air (i.e. threshold values that cannot be exceeded in the territory of EU) as regards the main air pollutants, (sulphur dioxide, nitrogen dioxide, nitrogen oxides, particulate matter, lead, benzene, carbon oxide and ozone) regulated by the Directive 1999/30/EC (EC,^1999^). Member states are obliged do indicate zones and urban areas for ambient air quality assessment and management, long-term tendency monitoring and making this information public. Where air quality is good, it is to be kept; where threshold values have been exceeded, proper action is to be taken. This directive is the first to include the objective related to air quality concerning particulate matter (PM_2.5_).

NO_2_, CO and PM exposure reduction targets for the protection of human health are included also in several WHO documents and guidelines (WHO 1987, 2000, 2006, 2012, 2013) (Table 3).

**Table 3:** Air pollutants concentrations and national and European standards (in μg/m^3^), based on WHO reports

Air pollutant	Time	WHO	EPA	EU	Poland	France	Germany	Great Britain
CO	10–15 min	100.000	–	–	–	–	–	–
	30 min	60,000	–	–	–	–	–	–
	1 h	30,000	40,000	–	–	–	–	–
	8 h	10,000	10,000	10,000	10,000	10,000	10,000	10,000
	24 h	–	–	–	–	–	–	–
	Year	–	–	–	–	–	–	–
NO_2_	30 min	–	–	–	–	–	200	–
	1 h	200	–	200 can be exceeded up to 18 times per year	200 can be exceeded up to 18 times per year	230 no longer than 0.2% of time	–	200 can be exceeded up to 18 times per year
	24 h	–	–	–	–	–	100	–
	Year	40	100	40	40	46	–	40

The air quality has significantly improved since guidelines started to be introduced in the European Community in the 1970s. EU has got three various legal mechanisms at its disposal that makes it possible to limit air pollution. Those are as follows: determining general air quality standards concerning the concentration of pollutants in the atmospheric air, determining state threshold values of joint pollutant emission and preparing legislation concerning specific sources of pollution, e.g. legislation on industrial emission control or on determination of standards concerning pollutant emission by vehicles, efficient energy use or fuel quality. This legislation is supplemented by strategies and measures allowing for promotion of environment protection and their inclusion in the transport and energy sector. A range of directives were adopted in order to limit pollution caused by road transport by way of determination of emission standards for various vehicle categories, e.g. passenger cars, light commercial vehicles, lorries, buses and motorcycles, and regulation of fuel quality. While CO_2_ emissions from new cars and vans are being successfully reduced under recent EU legislation, the heavy-duty vehicles (HDV) strategy, adopted in May 2014 (EU, 2014), is the EU’s first initiative to tackle such emissions from trucks, buses and coaches.

### Description of location of selected roads

The measurements were made in two stations located by the A4 motorway, in a section running near the city centre. The A4 motorway is a Polish section of international road E40 and E462 extending the German A4 motorway from Drezno; it runs across South Poland, from the border with Germany to a border checkpoint with Ukraine. In the territory of the Upper Silesian urban area, there are two key hubs: Gliwice-Sośnica, where A4 crosses the A1 motorway, and Katowice-Murckowska hub in Katowice, which connects the A4 motorway (Górnośląska Avenue) and national road no. 86. It has to be pointed out that A4 concentrates the transit and local traffic of a city of over 300 000 inhabitants and the Upper Silesian urban area of 5 million. The highway traffic intensity is 100,983 100983 vehicles per day (General Director for National Roads and Motorways, 2015).

ext measurements were made in a station located by national road no. 86, which runs through the city centre as well. National road no. 86 is the so-called fast traffic road. It connects the transit traffic between Katowice and Łódź and Warsaw and the local traffic as it runs through the centre of Częstochowa—a city of ca. 240 thousand. It is about 40 km long and is entirely located in the Silesian Voivodship. The expressway traffic intensity is 35,414 35414 vehicles per day (General Director for National Roads and Motorways, 2015). It is worth noting that the highway traffic intensity on both roads was similar to the same types of roads near big cities, in Germany, Netherlands or UK ( 30–150 30–150000 vehicles per day).

### Description of measurement points

The measurements were carried out in three measurement stations belonging to the Voivodship Environmental Protection Inspectorate.

**Map 1:**
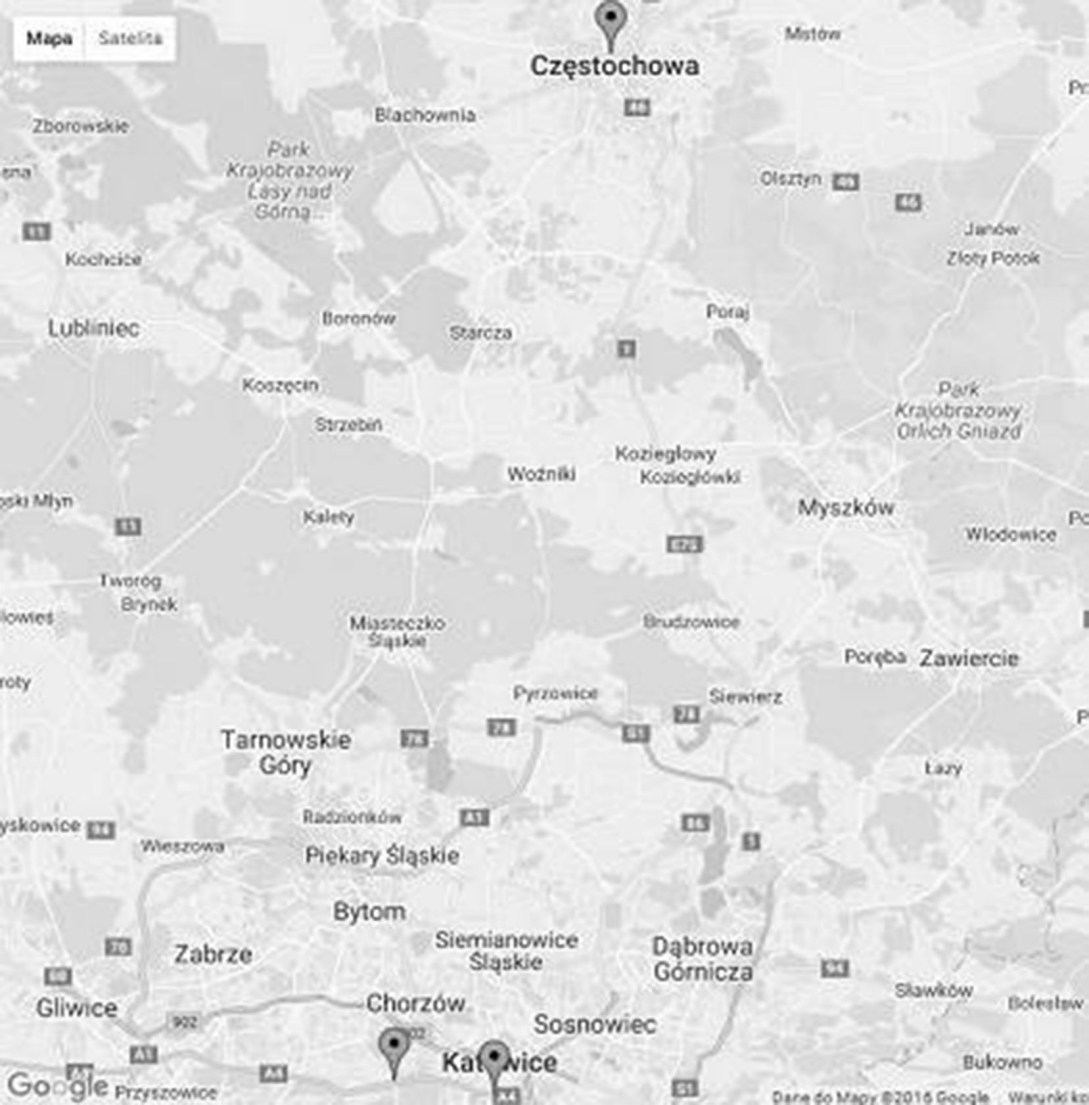
Map of air pollution monitoring sites in Chorzów, Katowice and Częstochowa in the Upper Silesian agglomeration

1. The first station was located in Chorzów Batory. Station type- communication. Monitoring type- automatic. Station type- stationary container. Distance from the nearest residential housing (m): 1000. Distance from the road (m): 7. The studies were conducted within the period from 2007/01/01 to 2010/12/07; the station was active till December 2010. The station had to be close because of the change of the road layout.
2. The second station is located in Katowice, Górnośląska Avenue, and it has functioned since January 2011. The studies were conducted from 2011/01/08 to 2014/05/31. Prevalent emission source: communication, measurement type: automatic. Main emission sources: fuel combustion processes—road transport. NO_2_ measurement method: Chemiluminescence’s. Measurement instrument: Environment S.A. Model AC32M NO_2_ Analyzer. CO measurement method: non-dispersive infrared spectroscopy (NDIR). Measurement instrument: Environment S.A. Model CO12M CO Analyzer. Method equivalence: reference method. Sampling time - number: 1. unit: hour. Sampling interval - number: 1, unit: hour. Sampling height (m): 3.7. Distance from the road (m): 10.
3. The third station is located in Częstochowa, Armii Krajowej Street. The measurements were carried out from 2007/01/01 to 2014/05/31. Measurement type: automatic. The prevalent emission source has a communication background. Main emission sources: fuel combustion processes—road transport. NO_2_ measurement method: Chemiluminescence’s. NO_2_ measuring instrument: Teledyne API T200 chemiluminescent NOx analyser. CO measurement method: non-dispersive infrared spectroscopy (NDIR). CO measuring instrument: Environment S.A. Model CO12M CO Analyzer. Method equivalence: reference method. Sampling time - number: 1, unit: hour. Sampling interval - number: 1. unit: hour. Sampling height (m): 3.7. Distance from the residential housing (m): 120. Distance from the road (m): 18 (Map 1).

### Nitrogen dioxide concentrations

#### The first station- Chorzów Batory

The dependence between the class of the air quality index (v. low and low) of average daily NO_2_ concentrations and year for the measured station was low (correlation ratio γ = 0.05). The average daily NO_2_ concentrations measured at the station corresponded more frequently to class 1 (very low) than to class 2 (low): 60.9 and 39.1, 53.7 and 46.3 and 58.0 and 42.0% as well as 67.0 and 33.0%, for year 2007, 2008, 2009 and 2010, respectively.

#### The second station- Katowice, Górnośląska avenue

The dependence between the class of the air quality index (v. low, low, medium) of average daily NO_2_ concentrations and year for the measured station was low (correlation ratio γ = 0.14). NO_2_ concentrations corresponding to class 1 (very low) and 2 (low) were much more frequent than those corresponding to class 3 (medium): 31.8, 63.6 and 4.6; 56.6, 43.4 and 0.0 and 68.7, 30.8 and 0.6% as well as 34.4, 59.6 and 6.0%, for year 2011, 2012, 2013 and 2014 (to May), respectively. No concentrations corresponding to class 4 (high) and 5 (very high) of the AQI were observed.

#### The third station- Częstochowa, Armii Krajowej street

**Fig. 1:**
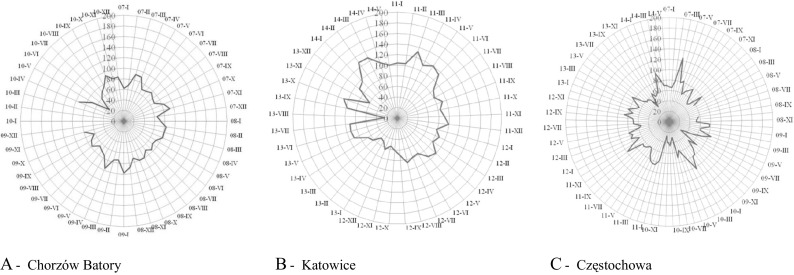
Radar chart of the highest values of the arithmetic means of NO_2_ (μg/m^3^) per 24 h measured by the automatic stations of the State Environmental Monitoring located in Chorzów Batory, Katowice and Częstochowa

**Fig. 2:**
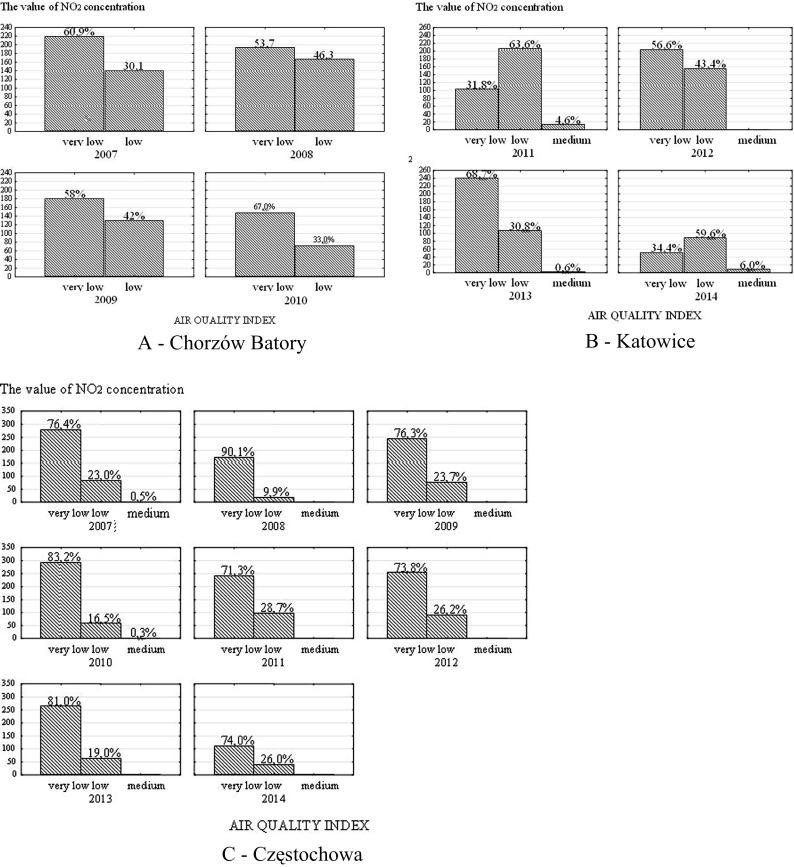
Histograms of empirical distributions of levels of air quality index to the arithmetic average values of NO_2_ per day measured in the automatic station of the State Environmental Monitoring in Chorzów Batory, Katowice and Częstochowa

The dependence between the average daily NO_2_ concentrations for the measured station and year was low (correlation ratio γ = 0.01, Cramér’s V = 0.1). The value of the average daily NO_2_ concentrations measured at the station “very low” or “low” were much more frequent than “medium”: 76.4, 23.0 and 0.6; 90.1, 9.9 and 0.0; 76.3, 23.7 and 0.0; 83.2, 16.5 and 0.3; 71.3, 28.7 and 0.0; 73.8, 26.2 and 0.0 and 81.0, 19.0 and 0.0% as well as 74.0, 26.0 and 0.0%, for year 2007, 2008, 2009, 2010, 2011; 2012; 2013 and 2014 (to May), respectively. No concentrations corresponding to class 4 (high) and 5 (very high) were noted. The value of the AQI—v. low—in relation to the average daily NO_2_ concentrations measured at the station, was most frequent for the year 2008: 76.4, 90.1, 76.3, 83.2, 71.3, 73.8, 81.0 and 74.0% for year 2007, 2008, 2009, 2010, 2011, 2012, 2013 and 2014 (to May), respectively. The value of the AQI—low—was most frequently observed for the year 2012: 23.0, 9.9, 23.7, 16.5, 28.7, 26.2, 19.0 and 26.0, whereas, the value of the AQI—medium—was most frequent for year 2007: 0.5, 0.0, 0.0, 0.3, 0.0, 0.0, 0.0 and 0.0% for year 2007, 2008, 2009, 2010, 2011, 2012, 2013 and 2014 (to May), respectively (Figs. 1 and 2).

No evidence was found of exceeding average daily values equal to the maximum allowable nitrogen dioxide concentration due to the protection of human health in the measurement area of all three stations.

### Carbon monoxide concentrations

#### The first station- Chorzów Batory

The dependence between the average daily CO concentrations and year for the measured station was very low (Kendall’s correlation ratio τ(τK) = 0.01). The values of average daily CO concentrations corresponded to class 1 (very low) of AQI below 1 mg/m^3^ were observed most frequently. No daily average values exceeding the maximum admissible CO concentration (8-h moving average) were noted in the examined period.

#### The second station- Katowice, Górnośląska avenue

The dependence between the average daily CO concentrations and year was low (Kendall’s correlation ratio τ(τK) = −0.13). The values of the average daily CO concentrations (for 8-h arithmetic moving average) measured in the stations corresponded to class 1 (very low) of the AQI.

#### The third station - Częstochowa, Armii Krajowej street

**Fig. 3:**
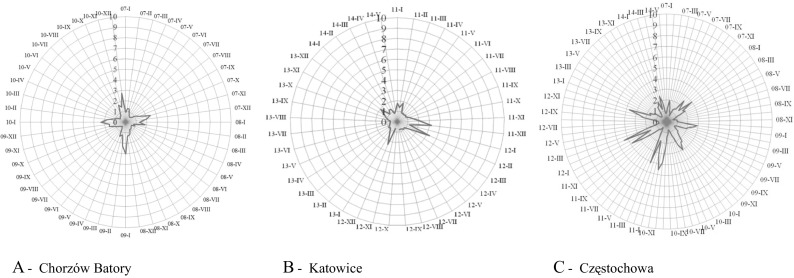
Radar chart of the highest values of the arithmetic means of CO (mg/m^3^) per 24 h measured by the automatic stations of the State Environmental Monitoring located in Chorzów Batory, Katowice and Częstochowa

**Fig. 4:**
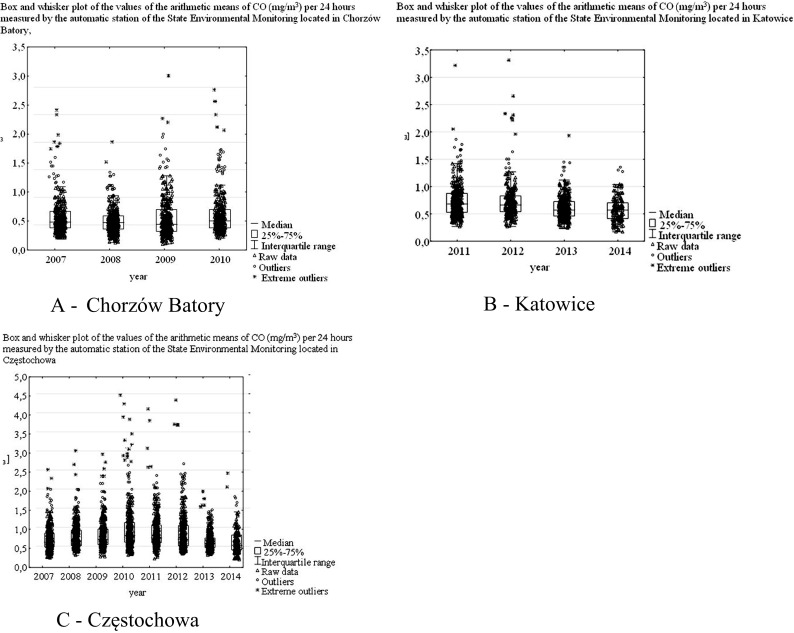
Box and whisker plot of the values of the arithmetic means of CO (mg/m^3^) per 24 h measured by the automatic station of the State Environmental Monitoring located in Chorzów Batory, Katowice and Częstochowa

The dependence between the average daily CO concentrations and year was very low (Kendall’s correlation ratio τ(τK) = −0.04). The value of the of CO concentration for the 8-h moving average corresponded to class 1 (very low) of AQI. The received values of parameters and shapes of empirical distributions as well as created box-plot allow to state that the most frequent CO concentrations were values below 1.5 mg/m^3^ (Table 4, Figs. 3 and 4).

**Table 4:** The values of selected parameters of CO concentration (average daily concentration) measured by three automatic stations of the State Environmental Monitoring: in Chorzów Batory Station (1); in Katowice, Górnośląska Avenue Station (2) and in Częstochowa, Armii Krajowej Street Station (3)

Year	N	Me	Min	Max	Q1	Q3	IQR	Vol. (%)	As
Station (1)									
2007	357	0.55	0.27	2.41	0.44	0.75	0.31	28.2	2.3
2008	359	0.54	0.18	1.87	0.42	0.66	0.24	22.7	1.4
2009	364	0.51	0.15	3.00	0.37	0.78	0.41	40.2	2.1
2010	331	0.57	0.26	2.76	0.44	0.78	0.34	30.3	2.2
Station (2)									
2011	352	0.68	0.27	3.22	0.52	0.88	0.36	26.5	2.3
2012	256	0.66	0.27	3.31	0.53	0.83	0.30	22.9	3.1
2013	351	0.57	0.24	1.93	0.45	0.73	0.28	24.6	1.4
2014	151	0.57	0.17	1.35	0.40	0.70	0.30	26.8	0.7
Station (3)									
2007	360	0.61	0.20	2.51	0.47	0.85	0.38	31.4	1.7
2008	363	0.64	0.26	3.00	0.50	0.92	0.42	33.2	2.1
2009	341	0.68	0.23	2.92	0.53	0.95	0.42	30.9	2.0
2010	365	0.78	0.26	4.52	0.59	1.12	0.53	34.0	2.5
2011	332	0.72	0.18	4.15	0.58	1.07	0.49	34.0	2.5
2012	361	0.68	0.31	4.39	0.50	1.05	0.55	40.4	2.5
2013	362	0.57	0.27	1.99	0.46	0.72	0.26	22.8	1.7
2014	149	0.52	0.15	2.47	0.39	0.80	0.41	39.4	1.8

*N* the sample size of examined general population considering the study statistical feature. *Me* median, *Min* minimum value of statistical feature, *Max* maximum value of statistical feature, *Q1* lower quartile (the first), *Q3* upper quartile (the third), *IQR* quartile deviation; *V*_*Q*_(*%*) coefficient variation, *Q=IQR/2* quartile deviation, *As* skewness

The daily average values corresponding to the maximum admissible CO concentration (8-h arithmetic moving average) in relation to the protection of human health were not exceeded in the measurement zone of the stations.

## Discussion

Transportation plays an important role in modern society, but its impact on air quality can have significant adverse effects on public health. Poland has experienced rapid motorization over the past 20 years, a trend that is likely to continue and a considerable development of road infrastructure, particularly in the recent decade; many new express roads and motorways, roads that run through city centres, ring roads and roads connecting cities were built. The growth in vehicles and the corresponding emissions create challenges to improving the urban air quality. Because many large neighbourhoods are located near heavy-traffic roads, it is essential to characterise near-road exposure to address concerns about public health and environmental justice. The construction of the new roads, especially like highways or expressways, very often led to large protests of local residents (i.e. the protest known as the Third Battle of Newbury some of the largest anti-road protests in European history, where around 7000 people demonstrated on the site of the bypass route and over 800 arrests being made, or the M11 link road protest was another major anti-road protest in Leytonstone, London, United Kingdom, in the early to mid-90s opposing the construction of the “A12 Hackney to M11 link road”, which was part of a significant local road scheme to connect traffic avoiding urban streets). It is worth noting that the development of both roads presented in this study was also accompanied with numerous protests of local communities. One of the arguments of the residents of a housing estate, located near A4, against the development of a new road was that they were most afraid of increased noise and pollution connected with high-density traffic. That is why, providing evidence-based information, which could serve policy-makers and also be accessible by the media and the general public, seems to be very important.

In an effort to reduce the impact of vehicle emissions on urban air quality, Poland has adopted a number of vehicle emission control strategies and policies over the last decades. These strategies and policies include adopting a series of European emission standards. These are classified into seven categories: emission control on new vehicles, emission control on in-use vehicles, fuel quality improvements, alternative fuel and advanced vehicles, economic policies, public transport and temporal traffic control measures. Many have proven to be successful, such as the Euro emission standards, unleaded gasoline and low sulphur fuel. In general, the adoption of stringent vehicle emission standards requires simultaneous fuel quality improvements. A close relationship between fuel quality and vehicle emissions has been confirmed by several studies (Wu et al., 2011). This is also expected due to newer vehicle technologies (Roy et al., 2009) introduced to meet new stringent regulations. Thanks to the emission standards for new vehicles as well as other controls, the fleet-average emission rates of CO, NO_2_ and PM_10_, by each major vehicle category, are decreasing over time.

## Conclusion

It is well known that motor vehicle exhaust is a significant source of air pollution and the most widely reported pollutants in vehicular exhaust include carbon monoxide and nitrogen oxide. While the evidence is considerable, it is not overwhelming and is weak in some areas; our study gives such an example. The results clearly indicate lack of hazards for general population health in terms of increased concentrations of CO and NO_2_ compounds that are closely related to high intensity car traffic found on selected motorways and speedways located near the city centres.
